# EC-WAMI: Event Camera-Based Pose Optimization in Remote Sensing and Wide-Area Motion Imagery

**DOI:** 10.3390/s24237493

**Published:** 2024-11-24

**Authors:** Isaac Nkrumah, Maryam Moshrefizadeh, Omar Tahri, Erik Blasch, Kannappan Palaniappan, Hadi AliAkbarpour

**Affiliations:** 1 Artificial Intelligence and Robotics Lab (AIRLab), Department of Computer Science, Saint Louis University, Saint Louis, MO 63103, USA; isaac.nkrumah@slu.edu (I.N.); mmoshrefizadeh@slu.edu (M.M.); 2 CNRS UMR 6303 ICB, Université de Bourgogne, 21078 Dijon, France; omar.tahri@u-bourgogne.fr; 3 MOVEJ Analytics, Fairborn, OH 45324, USA; 4 Electrical Engineering and Computer Science, University of Missouri, Columbia, MO 65211, USA; pal@missouri.edu

**Keywords:** remote sensing, WAMI, event camera, structure from motion, bundle adjustment, feature extraction, pose optimization

## Abstract

In this paper, we present *EC-WAMI*, the first successful application of neuromorphic *event cameras (ECs)* for Wide-Area Motion Imagery (WAMI) and Remote Sensing (RS), showcasing their potential for advancing Structure-from-Motion (SfM) and 3D reconstruction across diverse imaging scenarios. ECs, which detect asynchronous pixel-level *brightness changes*, offer key advantages over traditional frame-based sensors such as high temporal resolution, low power consumption, and resilience to dynamic lighting. These capabilities allow ECs to overcome challenges such as glare, uneven lighting, and low-light conditions that are common in aerial imaging and remote sensing, while also extending UAV flight endurance. To evaluate the effectiveness of ECs in WAMI, we simulate event data from RGB WAMI imagery and integrate them into SfM pipelines for camera pose optimization and 3D point cloud generation. Using two state-of-the-art SfM methods, namely, COLMAP and Bundle Adjustment for Sequential Imagery (BA4S), we show that although ECs do not capture scene content like traditional cameras, their spike-based events, which only measure *illumination changes*, allow for accurate camera pose recovery in WAMI scenarios even in low-framerate(5 fps) simulations. Our results indicate that while BA4S and COLMAP provide comparable accuracy, BA4S significantly outperforms COLMAP in terms of speed. Moreover, we evaluate different feature extraction methods, showing that the deep learning-based LIGHTGLUE descriptor consistently outperforms traditional handcrafted descriptors by providing improved reliability and accuracy of event-based SfM. These results highlight the broader potential of ECs in remote sensing, aerial imaging, and 3D reconstruction beyond conventional WAMI applications. Our dataset will be made available for public use.

## 1. Introduction

Remote Sensing (RS) serves as an effective method for continuous observation of a region by detecting its reflected or emitted signals from a distance. Wide-Area Motion Imagery (WAMI) [1] represents an RS approach designed to provide sustained and uninterrupted surveillance of vast geographic zones to enhance situational awareness [2] and object tracking [3]. WAMI is employed in such varied fields as urban development, traffic management, disaster assistance, emergency services, law enforcement, and surveillance [4,5]. Optimizing 3D camera poses by refining their positions and orientations in three-dimensional space and reconstructing the 3D structure of scenes using Structure-from-Motion (SfM) techniques are essential processes in preparing WAMI datasets for subsequent applications. SfM operates by detecting features across a series of images and tracking these features to estimate both the image structure and the camera motion [6,7,8]. Bundle Adjustment (BA) is an optimization process that refines the initial image position estimate by adjusting the camera parameters and point positions to minimize the reprojection error, which is the difference between the observed feature positions and the projected positions of the 3D points. SfM is widely used in many applications, including 3D reconstruction [7], RS [9], autonomous driving [10], robotics [11], and more.

Traditional cameras have played a significant role in SfM pipelines, particularly in the WAMI field. However, traditional RGB cameras suffer from inherent drawbacks such as motion blur, limited dynamic range, and restricted temporal resolution, which pose challenges in various applications [4,6]. In contrast, event cameras (ECs) are emerging bio-inspired vision sensors that offer exceptionally high temporal resolution and are able to capture *changes* in intensity at the pixel level as opposed to measuring their intensities [12]. Instead of capturing intensity images, ECs generate a continuous stream of events and encode critical information about the time, location, and polarity of these changes. ECs present revolutionary advantages compared to the paradigm of conventional frame-based cameras; with unique properties such as a dynamic range of 140 dB, resilience to non-uniform illumination, low latency, an ultra-high temporal resolution exceeding 100,000 fps, and power consumption of merely 1 mW, ECs offer significant advancements.

To the best of our knowledge, this is the first paper to employ event cameras for optimizing camera poses and performing SfM in RS and WAMI, establishing a novel contribution to the field. We study the integration of event data generated by ECs in two different SfM pipelines: COLMAP [7,13], and Bundle Adjustment for Sequential imagery (BA4S) [6,14,15]. COLMAP is a state-of-the-art open-source SfM software (ver 3.8) which includes two stages. The first stage is the traditional SfM part, which performs camera pose estimation and *sparse* 3D point reconstruction from unordered image sets. The second stage of COLMAP receives the recovered camera pose and sparse 3D point cloud obtained in stage 1 and performs a *dense* 3D point reconstruction. BA4S, another SfM pipeline, capitalizes on the temporal consistency found in video sequences. BA4S leverages sequential ordering of images to find feature correspondences for triangulation. It employs a statistical robust error function to handle outliers while avoiding the use of Random Sample Consensus (RANSAC), making the BA4S pipeline highly suitable for computationally expensive applications like WAMI. When combined with accessible but noisy metadata, BA4S achieves robust recovery of camera pose and sparse 3D point reconstruction, performing at a speed several times faster [6] than competing methods such as COLMAP [16]. Furthermore, we evaluate a variety of handcrafted features and recently developed deep learning-based feature in order to determine their effectiveness within EC-integrated SfM pipelines for WAMI. In summary, the main contributions of this work are as follows:

- We develop a robust pipeline for camera pose optimization based on EC-based WAMI datasets using SfM algorithms, specifically COLMAP and BA4S.
- We evaluate a variety of handcrafted features and recently developed deep learning-based features in order to determine their effectiveness within EC-integrated SfM pipelines for WAMI and RS.
- We share our dataset [17] to enable the replication of our findings and encourage further research on the use of ECs for camera pose optimization and 3D reconstruction in RS and WAMI applications.

## 2. Related Work

ECs have demonstrated significant promise in the field of 3D reconstruction, especially in dynamic environments where traditional frame-based cameras might encounter lighting challenges. One application in this area is the development of event-based multiview stereo techniques [18]. These methods harness the high temporal resolution of ECs to achieve real-time 3D reconstruction even under challenging lighting conditions and in fast-moving scenarios. Recent research by Ghosh and Gallego [19] delved into depth estimation using multiple ECs, introducing techniques for outlier rejection by fusing refocused events. Furthermore, ECs have been utilized for tracking the 6-DOF pose of a camera in a known environment using photometric 3D maps [20]. Several studies have explored the use of ECs in SfM and Visual Odometry (VO). A comprehensive exploration has been presented on event-driven feature detection and tracking specially tailored for Visual Simultaneous Localization and Mapping (SLAM), highlighting the evolving methodologies for ECs [21]. Xiao et al. [22] proposed a dense reconstruction pipeline specifically designed for ECs, while Kim [23] showed the feasibility of real-time visual SLAM using an EC, highlighting the advantages of high-speed and high-dynamic-range visual sensing. Furthermore, there have been significant contributions to data-driven feature tracking for ECs. For instance, Messikommer et al. [24] presented a data-driven feature tracker for ECs and introduced a novel frame attention module to improve tracking performance across various conditions. The asynchronous nature of ECs also poses challenges, particularly in data synchronization and fusion. These challenges have been addressed in various ways, including the development of specialized algorithms for pose estimation and 3D reconstruction using event-based data [25,26].

In summary, the use of ECs in SfM and VO applications presents both opportunities and challenges. While ECs offer significant advantages in terms of temporal resolution and dynamic range, their integration into existing algorithms and systems requires careful consideration and adaptation.

Feature extraction involves identifying and selecting distinctive and invariant points or features within images that can be reliably matched across different views. Expansive research has been accomplished to assess feature detectors and descriptors in computer vision for traditional cameras. Moreels and Perona’s [27] investigation into different combinations of feature detectors and descriptors concluded that pairing the Hessian-affine detector with the Scale-Invariant Feature Transform (SIFT) descriptor was most effective, particularly in handling perspective changes and varying lighting conditions. Meanwhile, Mikolajczyk and Schmid’s [28] work focused on testing a range of feature descriptors against the challenges of photometric and geometric alterations using the Oxford Affine Covariant Regions Dataset [29]. Their research highlighted that while performance can depend on the chosen feature detector, the SIFT descriptor generally stands out for its reliability. In another study, Heinly et al. [30] compared binary feature descriptors such as  BRIEF [31], BRISK [32], and ORB to floating-point descriptors such as SIFT. In addition, they used Speeded-Up Robust Features (SURF) with an expanded Oxford Dataset. They found that although binary descriptors offer a notable boost in speed, their effectiveness is subject to the type of image transformation encountered, with SIFT proving particularly adept at handling geometric distortions. Fan et al. [33] took a different approach by analyzing both handcrafted and learning-based features for use in 3D reconstruction tasks. They used DTU MVS datasets [34] that included a variety of scenes and materials as well as a large-scale SfM dataset containing images of landmarks from the internet. Recent advancements in learned descriptors have improved SfM performance; however, studies such [35] assessing DeepDesc have indicated that they sometimes fall short compared to established methods. A comprehensive evaluation of local feature descriptors for SfM and MVS [36] found that learned descriptors generally outperformed SIFT, although certain advanced handcrafted descriptors [37,38] showed comparable or superior efficacy in complex scenarios. Similarly, Gao et al. [39] presented a comprehensive evaluation across simulated city-scale aerial imagery, demonstrating that learned features significantly outperform traditional handcrafted features in terms of 3D reconstruction accuracy and feature track longevity. These studies collectively underscore the evolving landscape of feature extraction methods in computer vision, highlighting the diverse performance and applicability of different techniques across various scenarios.

**Figure 1:**
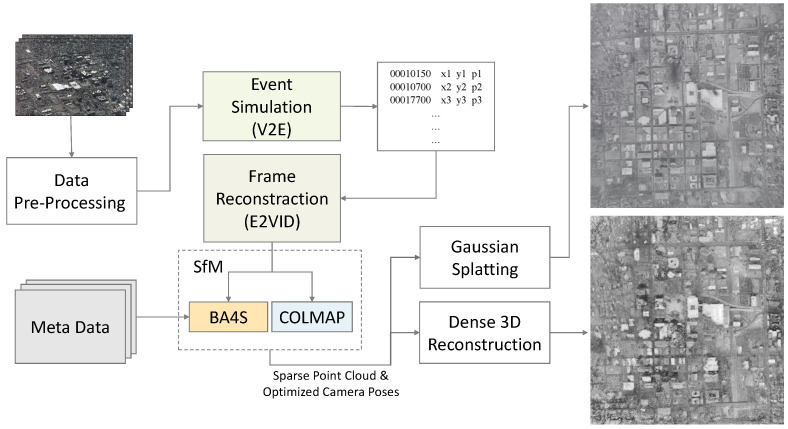
Block diagram of our EC-WAMI pipeline. RGB event data are simulated using an event simulator and frames are reconstructed with a frame reconstructor. The reconstructed frames are then fed into an SfM algorithm for camera pose optimization and 3D reconstruction.

Despite extensive research on ECs and WAMI, to the best of our knowledge the use of ECs for optimizing camera poses in WAMI has not been addressed prior to this work. We developed a robust pipeline for camera pose optimization based on EC-based WAMI datasets using SfM algorithms, specifically COLMAP and BA4S. Due to the scarcity of real-world event data, we simulated event data from WAMI RGB images, then extracted high-quality frames to facilitate accurate pose estimation and optimization (see Figure 1).

## 3. Method

In this section, we provide a comprehensive explanation of the methodology employed in this research. The overall process depicted in Figure 1 starts by simulating event data from RGB images using an event simulator. These simulated event data are then reconstructed into frames using a frame reconstruction algorithm. Finally, the reconstructed frames are input into a Structure from Motion (SfM) algorithm for camera pose optimization and 3D scene reconstruction. Each component is thoroughly explained, focusing on its function, implementation, and contribution to the research.

### 3.1. Neuromorphic Camera Model

Each pixel in a neuromorphic (event-based) camera (EC) operates independently in response to changes in logarithmic brightness, expressed as L=log(I) [12,40], where *I* is the intensity of light incident on the pixel. When the pixel brightness change exceeds a preset contrast threshold *C*, the pixel generates an event. For a specific event k at location x and time t, this can be described mathematically as

(1) $$\Delta L\left(x_{k},t_{k}\right)≐L\left(x_{k},t_{k}\right)-L\left(x_{k},t_{k}-\Delta t_{k}\right),$$

where $x_{k}$ is the pixel position for which event *k* occurs and $t_{k}$ is the timestamp for time *k*. An event occurs when the brightness change $\Delta L(x_{k},t_{k})$ equals $p_{k}C$, where $p_{k}$ is the polarity (+1 or −1) of the change, as expressed by

(2) $$\Delta L\left(x_{k},t_{k}\right)=p_{k}C.$$

The model in Equation (2) is idealized; in practice, sensor noise and other factors can affect the accuracy, for instance the variability in contrast sensitivity *C* due to pixel bias currents.

One key feature of ECs is their ability to approximate the temporal derivative of brightness. For small time intervals $\Delta t_{k}$, the brightness change can be related to the temporal derivative as follows:

(3) $$\frac{\partial L}{\partial t}\left(x_{k},t_{k}\right)\approx \frac{p_{k}C}{\Delta t_{k}}.$$

Each pixel in the EC responds to spatiotemporal brightness changes and outputs a stream of asynchronous events. A sequence of events is denoted $E=\{e_{1},e_{2},\ldots \}$, where each event $e_{n}=\left\{x_{n},y_{n},p_{n},t_{n}\right\}$ consists of the image coordinates $\left(x_{n},y_{n}\right)$, polarity $p_{n}$ in {−1,1}, and timestamp $t_{n}$ for n=1,dots,N as number of the event.

To optimize the camera pose, algorithms such as BA4S and COLMAP use frames rather than event streams. Event-to-frame reconstruction is crucial for these methods, which convert the event streams *E* into a sequence of images $I_{k}=\left\{I_{1},I_{2},\cdots \right\}$, where $I_{k}\in \left({\left[0,1\right]}^{W\times H}\right)$. The stream of events is divided into non-overlapping spatiotemporal windows $E_{i}$, each containing a fixed number of events. These reconstructed frames are used in SfM algorithms for camera pose optimization and 3D reconstruction, which are detailed in Section 3.2.

### 3.2. Camera Pose Optimization Using SfM and BA

Structure from Motion (SfM) is used to jointly optimize camera poses and reconstruct the 3D structure of a scene by triangulating point correspondences across multiple images [41]. The matched points are used to estimate the 3D position of scene points by finding the intersection of viewing rays passing through the corresponding image points. Various feature extraction and matching algorithms are available, as discussed in the next subsection.

Usually, the initial camera poses for triangulation are inaccurate. Bundle Adjustment (BA) is a common method for refining both camera poses and 3D points by minimizing the re-projection error (the difference between observed image points and their projected counterparts) from the estimated 3D points. Given *n* cameras and *m* 3D points, the BA optimization problem can be defined as follows:

(4) $$E=\min_{R_{i},t_{i},K_{i},X_{j}}\sum_{i=1}^{n}\sum_{j=1}^{m}{∥x_{ji}-g\left(X_{j},R_{i},t_{i},K_{i}\right)∥}^{2}$$

where $R_{i}$, $t_{i}$, and $K_{i}$ are the respective rotation matrix, translation vector, and intrinsic matrix for the *i*-th camera, $X_{j}$ is the *j*-th 3D point, and $x_{ji}$ denotes the 2D image coordinates of feature $X_{j}$ in the *i*-th camera.

**Figure 2:**
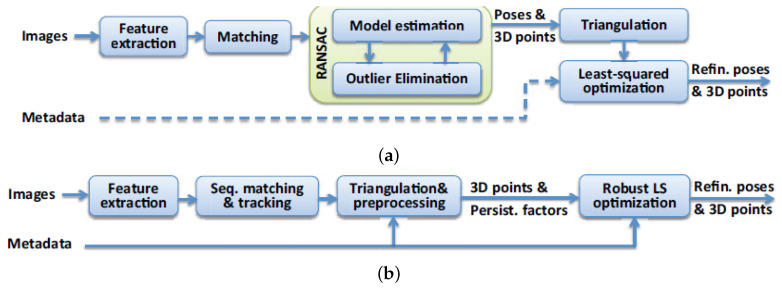
Conventional (e.g., COLMAP) versus BA4S SfM pipelines [15]. In the conventional SfM pipeline (**a**), camera poses and outliers are simultaneously estimated using RANSAC, and metadata may be used as extra constraints in optimization. In BA4S (**b**), camera metadata are used directly, and there is no model estimation, explicit outlier elimination, or RANSAC filtering of mismatches.

In practice, noise in the feature correspondences and estimated poses results in a nonzero re-projection error. The Levenberg–Marquardt (LM) algorithm [42] is commonly used to solve this BA problem by reducing the error through iterative optimization. BA4S improves upon traditional methods by applying a robust error function to mitigate the influence of outliers:

(5) $$\rho_{ji}\left(s_{ji},\gamma_{j},\mu_{F},\sigma_{F}\right)={\left(\frac{\gamma_{j}}{\mu_{F}+\sigma_{F}}\right)}^{2}\log\left(1+{\left(\frac{\mu_{F}+\sigma_{F}}{\gamma_{j}}\right)}^{2}s_{ji}^{2}\right)$$

where $s_{ji}={∥x_{ji}-g\left(X_{j},R_{i},t_{i},K_{i}\right)∥}^{2}$ is the projection residual and $\gamma_{j}$, $\mu_{F}$, and $\sigma_{F}$ are the persistency factor, mean, and standard deviation of the feature tracks, respectively [6]. The BA4S method effectively reduces outlier influence, leading to more accurate reconstructions. Figure 2 illustrates the differences between BA4S and conventional SFM pipelines.

### 3.3. Feature Detection and Description

Feature detection and matching are critical to the performance of SfM algorithms [6]. Feature detection involves identifying key points in images and describing them using feature descriptors. These descriptors are then used to match features across images by calculating the L2 Euclidean distance between descriptor vectors.

Below are some of the key feature descriptors used in this study:

- AKAZE  [43] detects keypoints using the Hessian matrix, with rotation-invariant descriptors based on intensity and gradient information.
- DCTF [44] utilizes the discrete cosine transform (DCTF) for illumination-robust descriptors, particularly in aerial imagery.
- ORB [45] is a binary descriptor using FAST detector and intensity centroids for keypoint orientation.
- SIFT [46], along with its GPU-based variant CUDASIFT, extracts keypoints using the difference of Gaussians and local gradient histograms.
- SURF [47], a faster alternative to SIFT, approximates the Laplacian of the Gaussian with a box filter.
- LIGHTGLUE [48], based on transformer architectures, adaptively matches local features based on image pair difficulty.

## 4. Experiments and Results

In this section, we discuss the process of data generation and camera pose optimization in WAMI scenarios using SfM pipelines. We evaluate the results both qualitatively and quantitatively. Due to the novelty of EC sensors, no real EC datasets are currently available for WAMI scenarios. Collecting real-world event data is costly and challenging. To develop an EC WAMI dataset, we used existing WAMI datasets from conventional RGB cameras and simulated event streams. The datasets included real RGB images from Albuquerque’s downtown area (ABQ-215) [6] and synthetic RGB imagery from Digital Imaging and Remote Sensing Image Generation—Rochester Institute of Technology (DIRSIG-RIT) [49]. Table 1 summarizes these two datasets, with detailed descriptions provided below.

**Figure 3:**
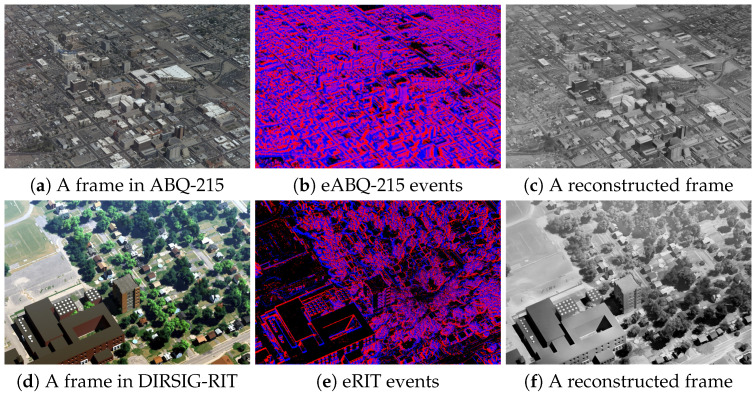
RGB sample frames in our WAMI datasets: Top, ABQ-215; Bottom, DIRSIG-RIT. The second column illustrates events simulated with Video-to-Event (V2E), while the third column shows the reconstructed frames from events simulated with Event-to-Video (E2VID).

**ABQ-215:** In our experiment, we utilized the ABQ-215 dataset (see Figure 3a for a sample frame), originally comprised of 215 frames each with a resolution of 6600 × 4400 pixels. To satisfy the computational requirements, the RGB images were downsampled, reducing their resolution to 1650 × 1100 pixels. This downsampling modification was essential in order to balance the tradeoff between computational efficiency and preservation of the critical visual details necessary for accurate analysis. The images were captured by an airborne traditional camera from an Above Ground Level (AGL) height of approximately 1.50 km while following an orbit radius of 2.5 km around the target area. This dataset also includes intrinsic parameters represented by the camera matrix K, which we scaled to match the downsampled images, and the extrinsic parameters of rotation matrix R and translation vector t.

**DIRSIG-RIT:** DIRSIG-RIT offers synthetic aerial images along with camera pose data. This synthetic imagery was created based on a hand-built 3D model of a suburban landscape. The simulated camera trajectory encompasses a complete circular orbit around the modeled scene. RIT contains 420 frames, each with dimensions of 1200 × 800 acquired with an AGL of approximately 0.9 km and an orbit radius of 1.40 km. A sample frame is shown in Figure 3d. Similar to ABQ-215, this dataset includes ground-truth metadata, consisting of intrinsic parameters represented by the camera matrix K and extrinsic parameters consisting of the rotation matrix R and translation vector t. No downsizing of the images was applied in this case, as the original image sizes were already adequate.

**Event Simulation using V2E:** The Video-to-Event (V2E) [40] algorithm is used for simulating realistic DVS event stream from video frames. V2E converts RGB video to a stream of events (see sample event frames in Figure 3b,e). The V2E algorithm was used to simulate event-based data in the absence of a physical DVS sensor. The method for converting video frames to DVS events involves converting RGB frames to luma, interpolating to increase temporal resolution, mapping luma to logarithmic intensity, applying a low-pass filter to model pixel bandwidth, and generating events based on intensity changes that exceed certain thresholds; the ON and OFF thresholds used in our experiment were 0.15, as expressed in Equations (1)–(3). This process ensures that realistic synthetic DVS events are produced for various applications, particularly when real-world event data are difficult to obtain.

**Frame Reconstruction from Event Streams:** After generating event streams using the V2E method, we applied a frame reconstruction method that aggregates the events to reconstruct a sequence of grayscale image frames, known as Event-to-Frame (E2F). Among existing E2F methods, we used E2VID by Rebecq et al. [12], which uses a Recurrent Neural Network (RNN) to reconstruct videos from a stream of events. The reconstruction of images from events involves translating a continuous stream of events into a sequence of images through a process that divides the incoming event stream into sequential non-overlapping spatiotemporal windows. Each window contains a fixed number of events. The stream processing approach utilizes an RNN that maintains and updates an internal state over time. For each sequence of events, a new image is generated using the network’s current state, then the state is updated accordingly. To reconstruct frames with higher quality, we used an average of two events per pixel for the ABQ-215 aerial imagery and one event per pixel for the RIT aerial imagery. Figure 3c,f shows example events and reconstructed frames from E2VID. The reconstructed frames are referred to as eABQ-215 and eRIT.

Simulating events from RGB video with low frame rates and reconstructing the simulated events to frames can be challenging, as some reconstructed frames may be blurry or distorted. To mitigate this, temporal resolution was increased by upsampling and interpolating the input video to ensure realistic event data for frame reconstruction [40]. Consequently, the number of reconstructed frames differed from that of the RGB images and associated metadata (the camera-intrinsic matrix **K**, rotation matrix **R**, and translation vector **t**). To match the number of reconstructed frames from the event-based data with the original RGB sequence, a regular sampling method was employed. However, we observed that the reconstructed frames generated by E2VID had irregular sampling intervals, which posed a challenge when assigning the available metadata of an original RGB frame to a corresponding reconstructed frame, resulting in the original RGB sequence and the E2VID reconstructed frames being out of sync. To find the closest reconstructed frame for each RGB image, we utilized the Structural Similarity Index Measure (SSIM) technique (see Algorithm 1). The SSIM evaluates the structural similarity between frames, allowing the most closely corresponding reconstructed frame to be identified for each original RGB frame. After establishing the closest correspondence between an original RGB frame and a reconstructed frame, the metadata from the matched RGB frame were assigned to the reconstructed frame. This SSIM-based selection ensured that the reconstructed frames were well-aligned to metadata, improving both alignment and positional accuracy.
**Algorithm 1** Find closest reconstructed images for RGB images and assign metadata1: **Input:** RGB image set *F* with metadata *M*, reconstructed image set $F^{\prime }$, window size *w*, length of $F^{\prime }$*n*2: **Output:** Set of closest reconstructed images $F^{\prime \prime }$ with assigned metadata $M^{\prime \prime }$3: Initialize start_index←04: **for** each RGB image $f_{i}$ in *F* **do**5:    **if** start_index≥n **then**6:        **Break**7:    **end if**8:    min_index←start_index9:    max_index← min(start_index+w−1, n−1)10:   $closest\_index\leftarrow \arg\max_{j}\;\left[SSIM\left(f_{i},f_{j}^{\prime }\right)\right]$, j∈[min_index,max_index]11:   $f_{i}^{\prime \prime }\leftarrow f_{closest\_index}^{\prime }$12:   $M_{i}^{\prime \prime }\leftarrow M_{i}$13:   start_index←closest_index+114:**end for**15:**Return** $F^{\prime \prime },M^{\prime \prime }$

### 4.1. Camera Pose Optimization Using SfM

In order to optimize the camera poses and generate sparse 3D point clouds from the sequence of images, we utilized BA4S and COLMAP as our SfM methods. In the case of ABQ-215 aerial imagery, the intrinsic parameters of the imprecise metadata were rescaled accordingly to match the dimensions of the reconstructed frames. For BA4S, we evaluated five handcrafted local feature descriptors (AKAZE, CUDASIFT, DCTF, ORB, SURF, SIFT) and one learned feature descriptor (LIGHTGLUE), while for COLMAP we primarily used the SIFT features [7,16,36]. Unlike BA4S, where only adjacent frames in a sequence are matched against each other, COLMAP exhaustively computes feature correspondences between all possible pairs of images in the sequence. Figure 2 highlights the differences between the BA4S and COLMAP SfM pipelines. In addition to sparse 3D points generated by the SfM algorithm, dense 3D point clouds can be reconstructed using an approach such as Multi-View Stereo (MVS) [13] or Gaussian splatting [50]. MVS is the second stage of the 3D reconstruction pipeline in the COLMAP software, (ver 3.8). Gaussian splatting is a recent approach for scene reconstruction that uses Gaussian primitives to represent the scene, enabling efficient and continuous 3D scene reconstruction from 2D images. Gaussian splatting diverges from traditional voxel-based or mesh-based approaches by using the Gaussians to represent the radiance and geometry of a scene in a smooth and continuous fashion, which can then be rendered and optimized to produce high-quality reconstructions.

All experiments were performed on a system with a 32-core Intel Core i9 and NVIDIA RTX 4090 GPU.

### 4.2. Evaluation Methods

To assess the feature accuracy within the SfM process, we computed the positional and angular errors for each camera using the recovered optimized camera poses. These recovered poses were then compared to ground-truth metadata derived from the traditional RGB aerial imagery. For the eABQ-215 dataset, the ground truth consisted of the refined and optimized camera poses obtained through BA4S on the corresponding RGB aerial dataset. To correct for any inherent offsets in SfM caused by the gauge freedom [41], the coordinate systems of the recovered poses were aligned to the ground-truth reference. The same evaluation metrics were applied to both BA4S and COLMAP. For a given camera *i* with a rotation matrix $R_{i}$ and translation vector $t_{i}$, the camera position $C_{i}$ is defined as follows:

(6) $$C_{i}=-R_{i}^{⊤}t_{i}$$

where the positions are normalized to make it scale-invariant and the percentage error $e_{c}$ is computed as

(7) $$e_{c_{i}}=\frac{∥\hat{C_{i}}-C_{i}{∥}_{2}}{∥C_{i}{∥}_{2}}\times 100,$$

where $\hat{C_{i}}$ is the estimated position of camera *i* using the reconstructed frame of the event data, $C_{i}$ is the ground truth location of camera *i*, and ${∥\cdot ∥}_{2}$ denotes the L2-norm representing the Euclidean distance.

The Root Mean Square Error (RMSE) offers a straightforward measure of accuracy for assessing the locations of feature points estimated by the SfM process. By calculating the square root of the average of the squares of the errors (the differences between the estimated and actual position locations), the RMSE quantifies how closely the SfM algorithm estimates the true positions of the camera in the scenes. The RMSE error for the camera pose is denoted by

(8) $$RMSE_{c}=\sqrt{\frac{1}{N}\sum_{i=1}^{N}e_{c_{i}}^{2}},$$

where N is the number of cameras. The camera angular error for camera *i* is then

(9) $$a_{c}\left(i\right)=\arccos\left[\frac{\left(\mathrm{trace}\left(R_{i}{\hat{R}}_{i}^{⊤}\right)-1\right)}{2}\right],$$

where $R_{i}$ is the rotation of the ground-truth pose of camera *i* and $\hat{R_{i}}$ is the rotation of the estimated camera pose of camera *i* Similar to the position error, the RMSE is calculated as shown below.

(10) $$RMSE_{a}=\sqrt{\frac{1}{N}\sum_{i=1}^{N}a_{c}^{2}\left(i\right)}$$

### 4.3. Discussion

**Figure 4:**
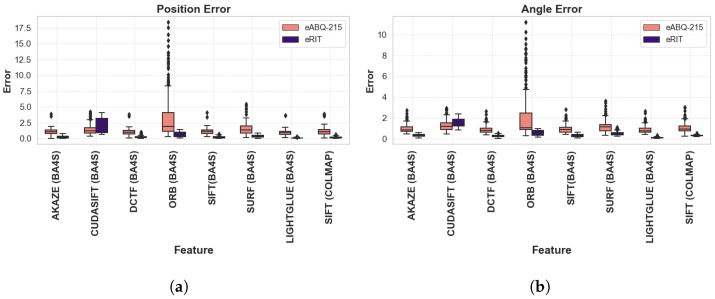
Errors of the recovered camera poses when using BA4S and COLMAP on the two aerial image sequences: (**a**) positional error (percentage in meters, using (7)) and (**b**) angular error (degrees, using (9)). LIGHTGLUE outperforms other feature descriptors in terms of both the positional and angular error metrics; in contrast, ORB shows comparatively lower performance and trails in terms of both measures.

As shown in Table 2, we evaluated eight different features using the BA4S and COLMAP SfM pipelines on the eABQ-215 and eRIT datasets. BA4S analyzes seven features, while COLMAP focuses on a single feature. ORB, which is widely used in Simultaneous Localization and Mapping (SLAM), provides the highest number of features on eABQ-215 and ranks second in the average number of features on eRIT. According to our metrics, AKAZE exhibits the highest average number of matches between pairs of images for eABQ-215. COLMAP (SIFT) has the highest average number of features, while ORB has the highest average number of matches between pairs of images on eRIT; however, the *LIGHTGLUE* learned feature descriptor performs best in almost all positional and angular error metrics across all datasets, with only DCTF outperforming it in terms of the RMSE angle error. This is consistent with the observation made by [39] that learned features outperform local handcrafted features on aerial imagery. LightGlue superior performance in our evaluations is due to its use of deep learning-based feature matching, which enables it to learn complex representations and handle the diverse challenges of WAMI datasets. Unlike traditional handcrafted methods, LightGlue uses attention mechanisms and global context awareness, allowing it to robustly match features across images with significant variations in scale, illumination, and viewpoint. Furthermore, its ability to adapt to noisy and degraded imagery combined with its learned invariance to photometric and geometric transformations makes it particularly well suited for the complex conditions present in aerial imagery. This adaptability and robustness are key reasons for its superior performance across all positional and angular error metrics [48]. Among the handcrafted features, DCTF performs competitively on both datasets, typically ranking just behind *LIGHTGLUE* in terms of the positional and angular errors metrics. This corroborates the findings by Gao et al. [44], who demonstrated that DCTF is capable of accurately recovering camera poses. The frequency domain operation of DCTF robustly captures essential spatial frequency information, making it invariant against common photometric transformations such as illumination changes and blurring. This enhances its reliability across varying conditions, especially for challenging reconstructed images. On the other hand, ORB did not perform well in terms of the positional and angular error metrics. Although ORB is optimized for fast feature detection and description, its simplicity limits its effectiveness in complex scenarios. Due to its use of binary descriptors, ORB may struggle to detect and match features accurately in environments with significant variations in texture, scale, or lighting. While its speed makes it suitable for real-time applications, its reduced robustness against scale changes and affine transformations often results in less reliable matches under challenging conditions. Therefore, ORB’s performance may be insufficient in scenarios requiring high accuracy and robust feature matching compared to more advanced methods such as SIFT and SURF. Another notable result is that the runtime for BA4S is more than 200 times faster compared to the runtime of COLMAP, since BA4S avoids the use of RANSAC and incorporates a persistency factor, as described in Section 3.2. Figure 4a,b summarizes the positional and angular errors on the two datasets.

**Figure 5:**
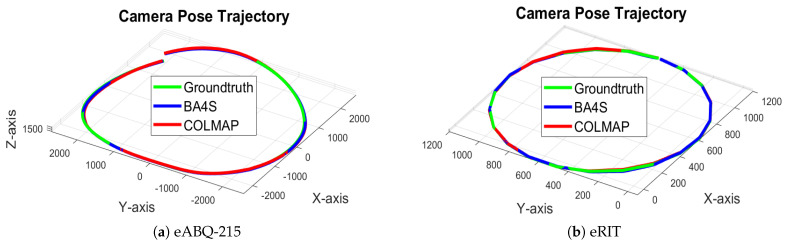
Recovered camera trajectories compared to ground truth for the eABQ-215 and eRIT datasets consisting of frames extracted from simulated event data. The recovered 3D trajectories closely match the ground truth.

**Figure 6:**
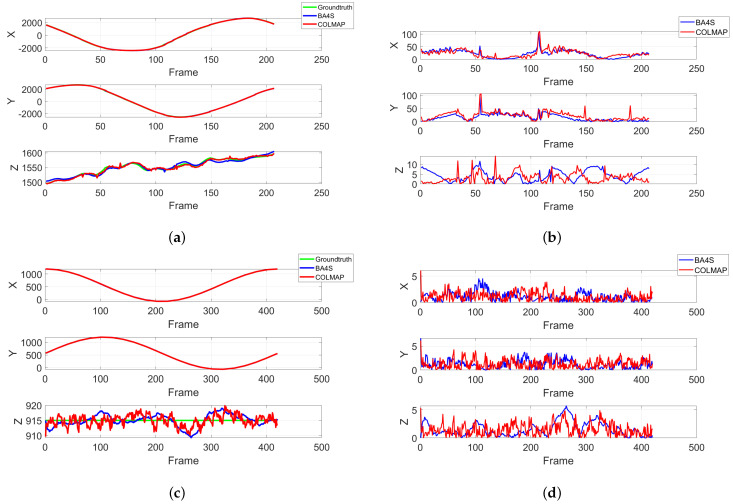
Recovered camera trajectories in 2D, showing the differences between BA4S and COLMAP on aerial images. The top row shows the trajectory and difference for the eABQ-215 dataset, while the second row illustrates the same results for the eRIT dataset. These graphs correspond to the 3D trajectories shown in Figure 5. BA4S displays a smoother trajectory, while COLMAP has a more jagged trajectory. There is a small difference between the ground truth and the optimized camera poses based on the reconstructed frames generated from simulated WAMI event data, demonstrating the effectiveness of our approach; Table 2 provides additional details. In (**a**), the optimized trajectory for the eABQ-215 dataset; (**b**), the difference between the optimized trajectory for eABQ-215 and the ground truth; (**c**), the optimized trajectory for the eRIT dataset; (**d**), the difference between the optimized trajectory for eRIT and the ground truth.

**Figure 7:**
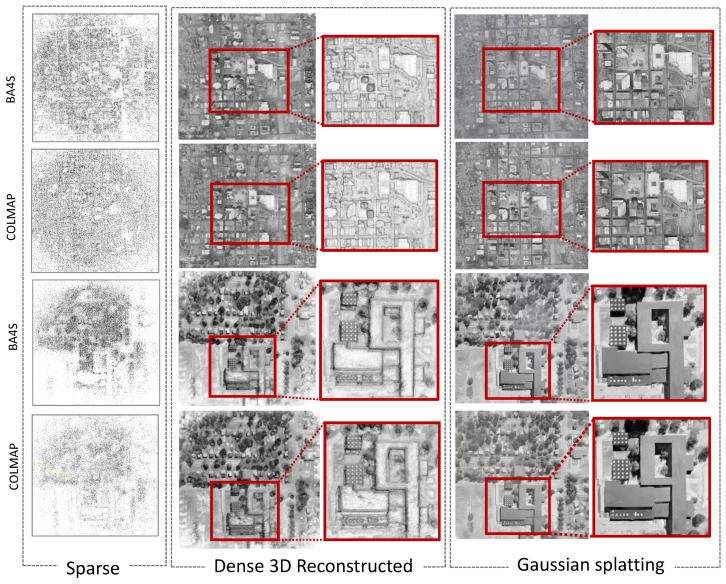
Sparse and dense 3D point clouds produced using the two SfM pipelines on the eABQ-215 and eRIT event camera datasets. The top two rows show the point clouds for eABQ-215 and the bottom two rows show the point clouds for eRIT. The results demonstrate that Gaussian splatting (GS) produces high-quality 3D scene reconstructions on both event datasets.

In addition, we conducted qualitative evaluations to assess the performance and effectiveness of BA4S and COLMAP. This included an analysis of the camera trajectories as well as an examination of both the sparse and dense 3D reconstructions. Figure 5 presents the camera trajectories from the SfM pipelines, while Figure 6 illustrates the differences between the ground truth and the optimized camera positions generated by BA4S and COLMAP. Utilizing SSIM to select those reconstructed frames that most closely matched each RGB image improved the metadata alignment, resulting in optimized camera positions that closely approximated the ground truth. Furthermore, the two SfM pipelines were both able to generate sparse 3D scenes. In addition to sparse 3D scene, dense point clouds can be reconstructed using approaches such as Multi-view Stereo (MVS) and Gaussian splatting. The point clouds of the eABQ-215 and eRIT datasets depicted in Figure 7 qualitatively demonstrate that Gaussian splatting produces the most accurate and detailed 3D scene reconstructions from the two event datasets. Overall, these results highlight the significant potential of using ECs in WAMI applications.

### 4.4. SfM Performance on Traditional Cameras in Challenging Illumination Scenarios

**Figure 8:**
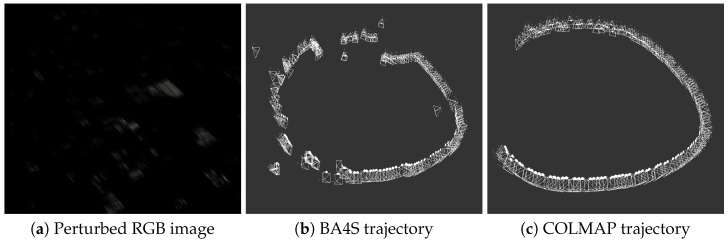
Camera pose recovery with a traditional RGB camera in a challenging illumination scenario: (**a**) shows a simulated RGB image with perturbations generated by applying techniques from [51] to the RGB image in Figure 3a, while (**b**,**c**) depict failed camera trajectory recovery when the perturbed traditional image was used as input for BA4S and COLMAP. These results underscore the limitations of traditional cameras in recovering pose under challenging illumination conditions.

Thus far, we have demonstrated the effectiveness of recovering 3D camera poses and scene structure using ECs. In this section, we aim to highlight the ineffectiveness of SfM methods for camera pose recovery when a traditional camera is used in a challenging scenario illumination. Unlike traditional cameras, ECs excel in capturing scene details under these conditions. Because real RGB images from applicable scenarios were unavailable, we simulated scenes captured by traditional cameras by applying the systematic image perturbation methods from [51]. Specifically, we used two corruption methods, namely, motion blur and low light, both of which were introduced in [51] and have been similarly applied in works such as [52]. These effects were applied simultaneously at varying levels of severity to simulate challenging conditions. To allow for a wide range of corruption, the source code was modified to accept negative values for severity. In the experiments, the brightness was first adjusted with a severity of −7, followed by motion blur with a severity of 8 at a 45° angle. Table 3 shows the output of this evaluation obtained with BA4S and COLMAP. Very few features were detected in the low light and motion blur scenario; as shown in Figure 8, the recovered trajectories include missing or bad camera poses. Moreover, CUDASIFT and COLMAP (SIFT) both failed to reconstruct and estimate the required number of camera poses for ABQ-215. Other similar experiments with ABQ-215 RGB images incorporating varying corruption severities provided similar or worse results.

## 5. Conclusions

In this study, we have demonstrated for the first time the application of event cameras in a WAMI and RS context and provided an evaluation of their effectiveness for camera pose optimization and 3D reconstruction using simulated event data. We integrated event streams into two state-of-the-art SfM pipelines, namely, COLMAP and BA4S, and conducted extensive experiments on the eABQ-215 and eRIT datasets. Our results show that despite the limited frame rate (5 fps) of the simulated RGB data, event cameras can enable robust and accurate camera pose recovery. BA4S significantly outperformed COLMAP in terms of computational efficiency, achieving camera pose optimization more than 200 times faster while maintaining comparable accuracy. We also evaluated several feature extraction methods, including traditional handcrafted descriptors and the LIGHTGLUE learned descriptor. Unsurprisingly, LIGHTGLUE demonstrated superior performance in both positional and angular accuracy across all datasets, reinforcing the benefits of deep learning-based feature matching in event-based SfM pipelines. Handcrafted features such as AKAZE and DCTF performed reasonably well, but lagged behind under more challenging conditions. Additionally, our qualitative analysis revealed that dense 3D reconstructions of event camera-based aerial imagery using camera poses optimized by SfM methods can provide remarkably detailed and accurate results.

Future directions include (1) collecting aerial imagery datasets using a real airborne event camera, (2) developing algorithms to process event streams directly in order to eliminate the need for frame reconstruction, (3) improving computational efficiency by integrating event data directly into the optimization engine, and (4) exploring deployment options such as containerized approaches [53].

## Figures and Tables

**Table 1:** Summary of aerial imagery datasets used to generate simulated events with Height Above Ground Level (AGL), circular orbit radius in km, and Average Ground Sampling Distance (GSD) in cm.

Dataset	ABQ-215	DIRSIG-RIT
Images	215	420
Image Size	1650 × 1100	1200 × 800
AGL (km)	1.50	0.90
Orbit Radius (km)	2.50	1.40
GSD (cm)	25	30

**Table 2:** Accuracy of estimated camera poses for the eABQ-215 and eRIT datasets using different features and SfM pipelines. The unit for the position error is the percentage in meters (using (7)), while the unit for for the angular error is degrees (using (9)). Here, Matches refer to the average number of matches per pair of images and Feat is the average number of features per image. It can be seen that BA4S has comparable accuracy to COLMAP along with an extreme advantage in speed, while the LIGHTGLUE learned feature outperforms the handcrafted features.

Dataset	SfM Method	Feature Name	Matches/ Feat. per Frame	BA Run Time (second)	RMSE Position	RMSE Angle	Median Position Error	Median Angle Error
eABQ-215	BA4S	AKAZE	4448/9406	14	1.1893	1.0135	1.0966	0.85239
	BA4S	CUDASIFT	530/2379	1	1.5497	1.3463	1.2428	1.1913
	BA4S	DCTF	4221/9406	13	1.0959	**0.92744**	0.98846	0.77838
	BA4S	ORB	2770/10,000	9	4.9935	3.1524	1.9064	1.0478
	BA4S	SIFT	2062/9995	7	1.2107	0.98387	1.158	0.86554
	BA4S	SURF	2471/9510	8	1.7369	1.2502	1.3818	1.1338
	BA4S	LIGHTGLUE	4129/5440	14	**1.0519**	0.93179	**0.94799**	**0.76476**
	COLMAP	SIFT	-/9579	4320	1.2819	1.1381	1.0537	0.92301
eRIT	BA4S	AKAZE	3857/5735	32	0.3199	0.33019	0.27832	0.30766
	BA4S	CUDASIFT	838/2295	6	2.3154	1.5905	1.6866	1.3773
	BA4S	DCTF	3525/5735	24	0.28635	0.26488	0.1885	0.25621
	BA4S	ORB	5031/10,000	33	0.74878	0.59767	0.59046	0.43641
	BA4S	SIFT	2568/8976	19	0.26101	0.34707	0.20188	0.3029
	BA4S	SURF	2393/5164	17	0.43692	0.53172	0.3761	0.44192
	BA4S	LIGHTGLUE	2642/3338	35	**0.10864**	**0.11043**	**0.08547**	**0.097197**
	COLMAP	SIFT	-/10,083	23372	0.22698	0.30015	0.17721	0.29782

**Table 3:** Evaluation of camera pose recovery simulating the use of a traditional (RGB) camera in a challenging scenario with significant illumination changes and fast motion. The images were generated from original RGB camera images (ABQ-215, Figure 3a) by adding motion blur and altering brightness (see Figure 8a). The results show poor performance for both SfM methods (COLMAP and BA4S), with most feature detection algorithms failing to identify enough features. These results highlight the inability of traditional cameras to recover the pose in challenging illumination scenarios.

Features	Matches/Feat. per Frame	RMSE Position	RMSE Angle
AKAZE	11/23	14.899	7.5709
CUDASIFT	0/0	-	-
DCTF	7/23	32.946	21.674
ORB	0/1	10.456	2.0263
SIFT	9/19	99.689	8.3719
SURF	7/20	96.094	13.219
COLMAP (SIFT)	110	-	-

## Data Availability

The data presented in this study are available at https://github.com/SLU-AIRLab/EC-WAMI.

## References

[1] Blasch E., Seetharaman G., Suddarth S., Palaniappan K., Chen G., Ling H., Basharat A. (2014). Summary of methods in wide-area motion imagery (WAMI). Proceedings of the Geospatial InfoFusion and Video Analytics IV and Motion Imagery for ISR and Situational Awareness II.

[2] Blasch E., Seetharaman G., Palaniappan K., Ling H., Chen G. (2012). Wide-area motion imagery (WAMI) exploitation tools for enhanced situation awareness. Proceedings of the 2012 IEEE Applied Imagery Pattern Recognition Workshop (AIPR).

[3] Ling H., Wu Y., Blasch E., Chen G., Lang H., Bai L. (2011). Evaluation of visual tracking in extremely low frame rate wide area motion imagery. Proceedings of the 14th International Conference on Information Fusion.

[4] Palaniappan K., Poostchi M., Aliakbarpour H., Viguier R., Fraser J., Bunyak F., Basharat A., Suddarth S., Blasch E., Rao R.M. (2016). Moving object detection for vehicle tracking in wide area motion imagery using 4d filtering. Proceedings of the 2016 23rd International Conference on Pattern Recognition (ICPR).

[5] Poostchi M., Aliakbarpour H., Viguier R., Bunyak F., Palaniappan K., Seetharaman G. Semantic depth map fusion for moving vehicle detection in aerial video. Proceedings of the IEEE Conference on Computer Vision and Pattern Recognition Workshops.

[6] AliAkbarpour H., Palaniappan K., Seetharaman G. (2017). Parallax-tolerant aerial image georegistration and efficient camera pose refinement—without piecewise homographies. IEEE Trans. Geosci. Remote Sens..

[7] Schonberger J.L., Frahm J.M. Structure-from-motion revisited. Proceedings of the IEEE conference on computer vision and pattern recognition.

[8] Wu C. (2013). Towards linear-time incremental structure from motion. Proceedings of the 2013 International Conference on 3D Vision-3DV.

[9] Turner D., Lucieer A., Watson C. (2012). An automated technique for generating georectified mosaics from ultra-high resolution unmanned aerial vehicle (UAV) imagery, based on structure from motion (SfM) point clouds. Remote Sens..

[10] Song S., Chandraker M. Robust scale estimation in real-time monocular SFM for autonomous driving. Proceedings of the IEEE Conference on Computer Vision and Pattern Recognition.

[11] Cavestany P., Rodriguez A.L., Martinez-Barbera H., Breckon T.P. (2015). Improved 3D sparse maps for high-performance SFM with low-cost omnidirectional robots. Proceedings of the 2015 IEEE International Conference on Image Processing (ICIP).

[12] Rebecq H., Ranftl R., Koltun V., Scaramuzza D. (2021). High Speed and High Dynamic Range Video with an Event Camera. IEEE Trans. Pattern Anal. Mach. Intell..

[13] Schönberger J.L., Zheng E., Frahm J.M., Pollefeys M. (2016). Pixelwise view selection for unstructured multi-view stereo. Proceedings of the Computer Vision–ECCV 2016: 14th European Conference.

[14] Seetharaman G., Palaniappan K., Akbarpour H.A. (2018). Method for Fast Camera Pose Refinement for Wide Area Motion Imagery. US Patent.

[15] AliAkbarpour H., Palaniappan K., Seetharaman G. Fast structure from motion for sequential and wide area motion imagery. Proceedings of the IEEE International Conference on Computer Vision Workshops.

[16] COLMAP. https://github.com/colmap/colmap.

[17] EC-WAMI-dataset. https://github.com/SLU-AIRLab/EC-WAMI.

[18] Rebecq H., Gallego G., Mueggler E., Scaramuzza D. (2018). EMVS: Event-based multi-view stereo—3D reconstruction with an event camera in real-time. Int. J. Comput. Vis..

[19] Ghosh S., Gallego G. (2022). Multi-Event-Camera Depth Estimation and Outlier Rejection by Refocused Events Fusion. Adv. Intell. Syst..

[20] Bryner S., Gallego G., Rebecq H., Scaramuzza D. (2019). Event-based, direct camera tracking from a photometric 3d map using nonlinear optimization. Proceedings of the 2019 International Conference on Robotics and Automation (ICRA).

[21] Alzugaray I. (2022). Event-Driven Feature Detection and Tracking for Visual SLAM. Ph.D. Thesis.

[22] Xiao K., Wang G., Chen Y., Nan J., Xie Y. (2022). Event-based dense reconstruction pipeline. Proceedings of the 2022 6th International Conference on Robotics and Automation Sciences (ICRAS).

[23] Kim H. (2018). Real-Time Visual SLAM with an Event Camera. Ph.D. Thesis.

[24] Messikommer N., Fang C., Gehrig M., Scaramuzza D. Data-driven feature tracking for event cameras. Proceedings of the IEEE/CVF Conference on Computer Vision and Pattern Recognition.

[25] Bryson M., Johnson-Roberson M., Murphy R.J., Bongiorno D. (2013). Kite aerial photography for low-cost, ultra-high spatial resolution multi-spectral mapping of intertidal landscapes. PLoS ONE.

[26] Eltner A., Kaiser A., Castillo C., Rock G., Neugirg F., Abellán A. (2016). Image-based surface reconstruction in geomorphometry–merits, limits and developments. Earth Surf. Dyn..

[27] Moreels P., Perona P. (2007). Evaluation of features detectors and descriptors based on 3D objects. Int. J. Comput. Vis..

[28] Mikolajczyk K., Schmid C. (2005). A performance evaluation of local descriptors. IEEE Trans. Pattern Anal. Mach. Intell..

[29] Mikolajczyk K., Schmid C. (2004). Comparison of affine-invariant local detectors and descriptors. Proceedings of the 2004 12th European Signal Processing Conference.

[30] Heinly J., Dunn E., Frahm J.M. (2012). Comparative evaluation of binary features. Proceedings of the Computer Vision–ECCV 2012: 12th European Conference on Computer Vision.

[31] Calonder M., Lepetit V., Strecha C., Fua P. (2010). Brief: Binary robust independent elementary features. Proceedings of the Computer Vision–ECCV 2010: 11th European Conference on Computer Vision.

[32] Leutenegger S., Chli M., Siegwart R.Y. (2011). BRISK: Binary robust invariant scalable keypoints. Proceedings of the 2011 International Conference on Computer Vision.

[33] Fan B., Kong Q., Wang X., Wang Z., Xiang S., Pan C., Fua P. (2019). A performance evaluation of local features for image-based 3D reconstruction. IEEE Trans. Image Process..

[34] Jensen R., Dahl A., Vogiatzis G., Tola E., Aanæs H. Large scale multi-view stereopsis evaluation. Proceedings of the IEEE Conference on Computer Vision and Pattern Recognition.

[35] Simo-Serra E., Trulls E., Ferraz L., Kokkinos I., Fua P., Moreno-Noguer F. Discriminative learning of deep convolutional feature point descriptors. Proceedings of the IEEE International Conference on Computer Vision.

[36] Schonberger J.L., Hardmeier H., Sattler T., Pollefeys M. Comparative evaluation of hand-crafted and learned local features. Proceedings of the IEEE Conference on Computer Vision and Pattern Recognition.

[37] Bursuc A., Tolias G., Jégou H. Kernel local descriptors with implicit rotation matching. Proceedings of the 5th ACM on International Conference on Multimedia Retrieval.

[38] Dong J., Soatto S. Domain-size pooling in local descriptors: DSP-SIFT. Proceedings of the IEEE Conference on Computer Vision and Pattern Recognition.

[39] Gao K., Aliakbarpour H., Fraser J., Nouduri K., Bunyak F., Massaro R., Seetharaman G., Palaniappan K. (2020). Local feature performance evaluation for structure-from-motion and multi-view stereo using simulated city-scale aerial imagery. IEEE Sensors J..

[40] Hu Y., Liu S.C., Delbruck T. v2e: From video frames to realistic DVS events. Proceedings of the IEEE/CVF Conference on Computer Vision and Pattern Recognition.

[41] Hartley R., Zisserman A. (2003). Multiple View Geometry in Computer Vision.

[42] Jeong Y., Nister D., Steedly D., Szeliski R., Kweon I.S. (2011). Pushing the envelope of modern methods for bundle adjustment. IEEE Trans. Pattern Anal. Mach. Intell..

[43] Alcantarilla P.F., Solutions T. (2011). Fast explicit diffusion for accelerated features in nonlinear scale spaces. IEEE Trans. Patt. Anal. Mach. Intell.

[44] Gao K., Aliakbarpour H., Seetharaman G., Palaniappan K. (2021). DCT-Based Local Descriptor for Robust Matching and Feature Tracking in Wide Area Motion Imagery. IEEE Geosci. Remote Sens. Lett..

[45] Rublee E., Rabaud V., Konolige K., Bradski G. (2011). ORB: An efficient alternative to SIFT or SURF. Proceedings of the 2011 International Conference on Computer Vision.

[46] Lowe D.G. (2004). Distinctive image features from scale-invariant keypoints. Int. J. Comput. Vis..

[47] Bay H. (2006). Surf: Speeded up robust features. Computer Vision—ECCV.

[48] Lindenberger P., Sarlin P., Pollefeys M. (2023). Lightglue: Local feature matching at light speed. arxiv.

[49] Nilosek D., Walvoord D.J., Salvaggio C. (2014). Assessing geoaccuracy of structure from motion point clouds from long-range image collections. Opt. Eng..

[50] Kerbl B., Kopanas G., Leimkühler T., Drettakis G. (2023). 3D Gaussian Splatting for Real-Time Radiance Field Rendering. ACM Trans. Graph..

[51] Hendrycks D., Dietterich T. (2019). Benchmarking neural network robustness to common corruptions and perturbations. arxiv.

[52] Tomy A., Paigwar A., Mann K.S., Renzaglia A., Laugier C. (2022). Fusing event-based and rgb camera for robust object detection in adverse conditions. Proceedings of the 2022 International Conference on Robotics and Automation (ICRA).

[53] Wu R., Liu B., Chen Y., Blasch E., Ling H., Chen G. (2017). A container-based elastic cloud architecture for pseudo real-time exploitation of wide area motion imagery (wami) stream. J. Signal Process. Syst..

